# Altered Response Pattern following AZD5582 Treatment of SIV-Infected, ART-Suppressed Rhesus Macaque Infants

**DOI:** 10.1128/jvi.01699-21

**Published:** 2022-03-16

**Authors:** Katherine M. Bricker, Veronica Obregon-Perko, Brianna Williams, Danielle Oliver, Ferzan Uddin, Margaret Neja, Louis Hopkins, Amir Dashti, Sherrie Jean, Jennifer S. Wood, Stephanie Ehnert, Shan Liang, Thomas Vanderford, Gregory K. Tharp, Steven E. Bosinger, Amanda P. Schauer, Maud Mavigner, Mackenzie L. Cottrell, David Margolis, Richard M. Dunham, Ann Chahroudi

**Affiliations:** a Department of Pediatrics, Emory University School of Medicine, Atlanta, Georgia, USA; b Yerkes National Primate Research Center, Emory University grid.471395.dgrid.189967.8grid.471395.dgrid.189967.8 , Atlanta, Georgia, USA; c Department of Pathology and Laboratory Medicine, Emory University School of Medicine, Atlanta, Georgia, USA; d Division of Pharmacotherapy and Experimental Therapeutics, UNC Eshelman School of Pharmacy, University of North Carolina at Chapel Hill, Chapel Hill, North Carolina, USA; e Center for Childhood Infections and Vaccines of Children’s Healthcare of Atlanta and Emory University grid.471395.dgrid.189967.8grid.471395.dgrid.189967.8 , Atlanta, Georgia, USA; f Division of Infectious Diseases, Department of Medicine, University of North Carolina at Chapel Hill, Chapel Hill, North Carolina, USA; g Center for AIDS Research, University of North Carolina at Chapel Hill, Chapel Hill, North Carolina, USA; h UNC HIV Cure Center, University of North Carolina at Chapel Hill, Chapel Hill, North Carolina, USA; i Department of Microbiology and Immunology, School of Medicine, University of North Carolina at Chapel Hill, Chapel Hill, North Carolina, USA; j Department of Epidemiology, Gillings School of Public Health, University of North Carolina at Chapel Hill, Chapel Hill, North Carolina, USA; k Qura Therapeutics, Chapel Hill, Research Triangle Park, North Carolina, USA; l HIV Drug Discovery, ViiV Healthcare, Research Triangle Park, North Carolina, USA; Ulm University Medical Center

**Keywords:** HIV, HIV cure, NHP, pediatric HIV, SIV

## Abstract

The “shock and kill” strategy for HIV-1 cure incorporates latency-reversing agents (LRA) in combination with interventions that aid the host immune system in clearing virally reactivated cells. LRAs have not yet been investigated in pediatric clinical or preclinical studies. Here, we evaluated an inhibitor of apoptosis protein (IAP) inhibitor (IAPi), AZD5582, that activates the noncanonical NF-κB (ncNF-κB) signaling pathway to reverse latency. Ten weekly doses of AZD5582 were intravenously administered at 0.1 mg/kg to rhesus macaque (RM) infants orally infected with SIV_mac251_ at 4 weeks of age and treated with a triple ART regimen for over 1 year. During AZD5582 treatment, on-ART viremia above the limit of detection (LOD, 60 copies/mL) was observed in 5/8 infant RMs starting at 3 days post-dose 4 and peaking at 771 copies/mL. Of the 135 measurements during AZD5582 treatment in these 5 RM infants, only 8 were above the LOD (6%), lower than the 46% we have previously reported in adult RMs. Pharmacokinetic analysis of plasma AZD5582 levels revealed a lower Cmax in treated infants compared to adults (294 ng/mL versus 802 ng/mL). RNA-Sequencing of CD4^+^ T cells comparing pre- and post-AZD5582 dosing showed many genes that were similarly upregulated in infants and adults, but the expression of key ncNF-κB genes, including *NFKB2* and *RELB*, was significantly higher in adult RMs. Our results suggest that dosing modifications for this latency reversal approach may be necessary to maximize virus reactivation in the pediatric setting for successful “shock and kill” strategies.

**IMPORTANCE** While antiretroviral therapy (ART) has improved HIV-1 disease outcome and reduced transmission, interruption of ART results in rapid viral rebound due to the persistent latent reservoir. Interventions to reduce the viral reservoir are of critical importance, especially for children who must adhere to lifelong ART to prevent disease progression. Here, we used our previously established pediatric nonhuman primate model of oral SIV infection to evaluate AZD5582, identified as a potent latency-reversing agent in adult macaques, in the controlled setting of daily ART. We demonstrated the safety of the IAPi AZD5582 and evaluate the pharmacokinetics and pharmacodynamics of repeated dosing. The response to AZD5582 in macaque infants differed from what we previously showed in adult macaques with weaker latency reversal in infants, likely due to altered pharmacokinetics and less inducibility of infant CD4^+^ T cells. These data supported the contention that HIV-1 cure strategies for children are best evaluated using pediatric model systems.

## INTRODUCTION

Despite increased access to interventions to prevent perinatal transmission of HIV-1, pediatric HIV-1 continues to be a global health crisis with 1.7 million children infected worldwide and 150,000 new pediatric cases annually (1). Most new infections occur postnatally through the breastmilk transmission route (2). Antiretroviral therapy (ART) has dramatically improved disease outcomes and reduced mortality but does not eliminate the long-lived viral reservoir established during acute infection (3–6). Strategies to reduce or eliminate the viral reservoir to allow periods of ART-free remission toward a long-term functional or sterilizing cure would greatly benefit children who must adhere to lifelong ART to prevent progression to AIDS.

HIV-1 infection differs in children and adults, with children experiencing higher peak and set-point viremia, slower decline to viral set point, and lower median survival in the absence of ART (7). Additionally, the developing immune system yields a unique environment that may influence both the pediatric HIV-1 reservoir and the impact of HIV-1 cure strategies on this population. For these reasons, we advocate for HIV-1 cure strategies to be investigated specifically in children. The use of a relevant pediatric animal model can provide important safety and efficacy information necessary to bring experimental therapeutics to pediatric trials. Simian immunodeficiency virus (SIV) infection in the rhesus macaque (RM) has long been established as a robust animal model for HIV-1 and has been used extensively to inform HIV-1 cure strategies (6, 8, 9). Previously published work from our laboratory has demonstrated that oral SIV and simian-human immunodeficiency virus (SHIV) infection of infant RMs can simulate postnatal HIV-1 infection through breastfeeding with ART-mediated suppression of viremia then permitting the study of virus persistence (10, 11). Through this model, we identified naive CD4^+^ T cells as a significant contributor to the viral reservoir in both SIV and SHIV infection of infant RMs (10, 11) and tested therapeutic vaccination in combination with TLR-7 stimulation to promote anti-SIV immune responses (12). This preclinical model provides further opportunities to test important hypotheses regarding viral reservoirs, infant immunity, and remission/eradication strategies.

The “shock and kill” HIV-1 cure strategy aims to “shock” virally infected cells out of a state of latency using a latency-reversing agent (LRA) to induce the production of viral RNA and proteins while introducing a “kill” therapeutic agent to aid the immune system in the clearance of infected, reactivated cells (13–16). Performing such interventions in the controlled setting of ART prevents new rounds of infection in uninfected cells while allowing intervention-mediated clearance of the existing reservoir. There has been extensive research into potential LRAs in preclinical and clinical trials; however, no LRA has yet been evaluated in infants or children. In recent work, we identified an inhibitor of apoptosis protein (IAP) inhibitor (IAPi), AZD5582 (also called a SMAC mimetic), as an effective LRA that induced reactivation of the viral reservoir in SIV-infected adult RMs treated with ART (17). Importantly, AZD5582 reactivates cells by targeting the noncanonical NF-κB pathway (ncNF-κB), reducing off-target effects and improving safety over agents that activate canonical NF-κB cell signaling.

In the present study, we evaluated AZD5582 in our preclinical model of SIV-infected ART-suppressed RM infants. We reported induction of on-ART viremia in 63% of AZD5582-treated infant RMs, but considerably reduced frequency of latency reversal compared to adult RMs. Transcriptomic analyses revealed significant differences in gene expression induced by AZD5582 in CD4^+^ T cells from infant and adult RMs. An altered pharmacokinetic profile of plasma AZD5582 in infant RMs was also observed, which may explain the dampened latency reversal. This study provides a novel understanding of how an IAPi interacts with the pediatric viral reservoir and developing immune system and suggests that optimization of LRA dosing may be crucial for “shock and kill” strategies to be effective in children.

## RESULTS

### *In vivo* experimental design and treatments.

**FIG 1:**
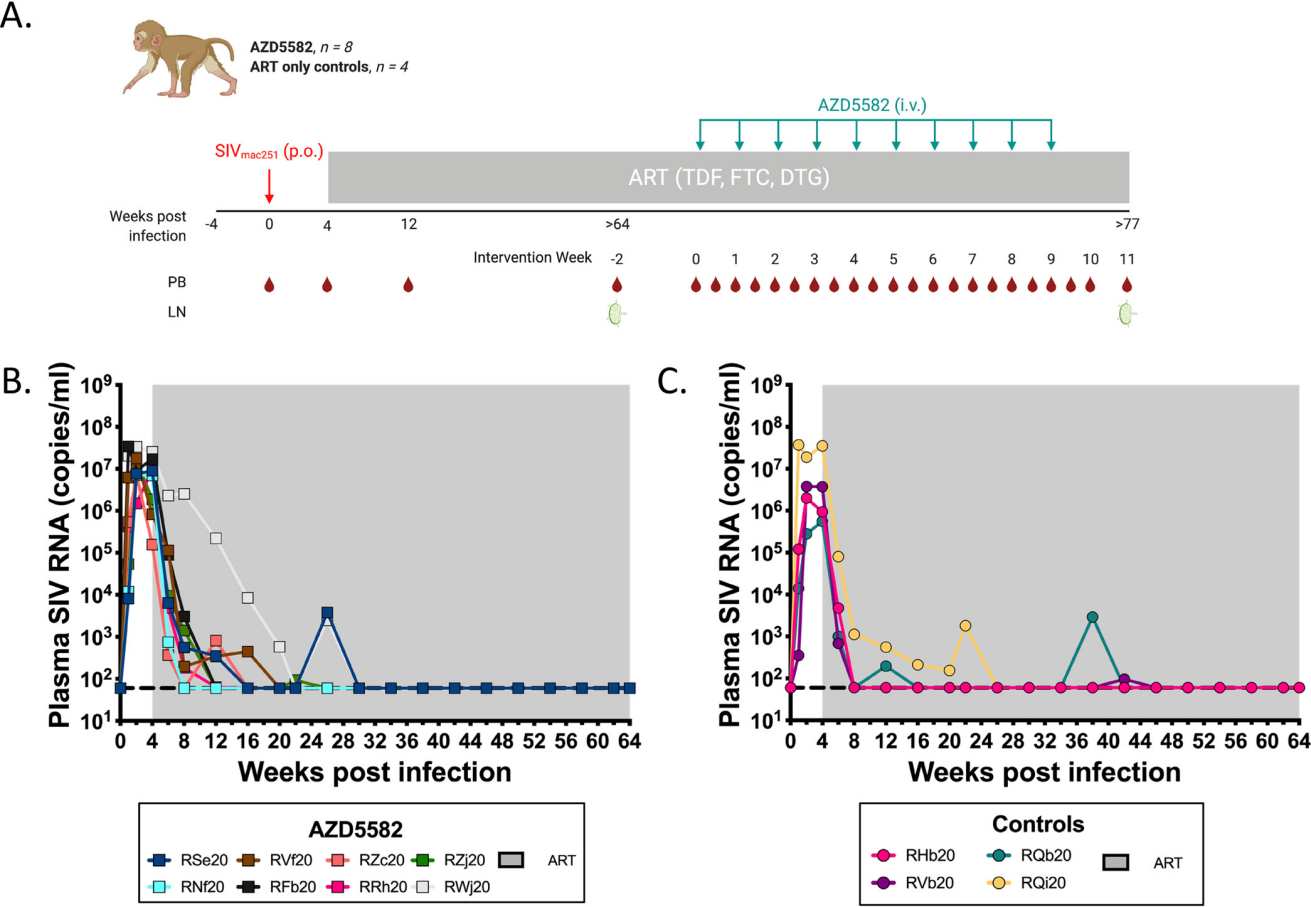
Experimental design and response to ART in SIV-infected infant RMs. (A) Schematic of the study design. Twelve infant RMs were infected orally with 10^5^ TCID50 SIV_mac251_ (day 0) and starting at 4 weeks postinfection were treated with combination ART (TDF, FTC, DTG). Eight animals received 10 doses of AZD5582 (0.1 mg/kg, i.v. infusion) at the indicated time points. The remaining 4 animals served as ART-treated controls. Peripheral blood (PB) and lymph node (LN) biopsy specimens were collected at the indicated time points. Longitudinal analysis of plasma SIV RNA levels pre-ART and during ART (but before AZD5582 treatment) in (B) AZD5582 and (C) control groups. The shaded area represents the period of ART treatment. The dashed line represents the limit of detection of the assay.

In this study, we sought to evaluate the impact of *in vivo* AZD5582 administration in SIV-infected, ART-suppressed infant rhesus macaques. Twelve Indian-origin RMs (four males and eight females) were selected for this study. RMs were confirmed negative for the MHC haplotypes (Mamu-B*08 and Mamu-B*17) associated with natural control of SIV replication. The time course of the experimental design and interventions used are shown in Fig. 1A. All RMs were exposed to two consecutive doses of 10^5^ TCID_50_ (50% tissue culture infective doses) SIV_mac251_ by oral administration at approximately 4 weeks of age (range: 3.1 to 7.4 wk; mean: 4.45 wk). As breastmilk acquisition of HIV-1 is unlikely to be followed by very early ART initiation (i.e., within hours or days), we started daily ART in all RMs at 4 weeks after SIV infection. The ART regimen (tenofovir, TDF; emtricitabine, FTC; dolutegravir, DTG) was administered as a single dose coformulation once daily by subcutaneous injection, as described previously (10–12), throughout the experimental time course (indicated by gray shading in Fig. 1A). ART was effective at suppressing SIV RNA in plasma below the limit of detection (LOD; 60 copies/mL) in all RMs (Fig. 1B and C). As observed in children with HIV-1 (18–20), time to viral suppression was variable, ranging from 4 to 26 weeks (median = 12 wk) with some infants showing transient blips of viremia shortly following viral suppression.

Before the AZD5582 treatment phase of the experiment, two groups of RMs (control, *n* = 4; experimental [‘AZD5582’], *n* = 8) were balanced for sex, age at infection, CD4^+^ T cell frequency at ART initiation, peak viral load, and area under the curve (AUC) of pre-ART viremia (Table 1). After over 1 year of daily ART (range: 66 to 70 wk), 8 RMs received 10 weekly doses of AZD5582 at 0.1 mg/kg by intravenous (i.v.) infusion (Fig. 1A). This dose is tolerated in adult RMs and effective in reversing latency (17). The remaining four RMs served as ART-treated controls. Peripheral blood (PB) and lymph node (LN) biopsy specimens were collected longitudinally at the time points indicated in Fig. 1A. AZD5582 was well tolerated in infant macaques, without clinical adverse events throughout the intervention phase of the study.

**TABLE 1:** Parameters used to select experimental and control groups

Group	ID	Sex	Age at infection, wks	CD4 frequency, ART initiation	Peak PVL, pre-ART	AUC pVL, pre-ART
AZD5582	RVf20	F	3.1	47.9%	1.76 × 10^7^	3.34 × 10^7^
	RNf20	F	3.3	35.6%	7.13 × 10^6^	1.77 × 10^7^
	RSe20	F	3.9	25.5%	9.01 × 10^6^	2.05 × 10^7^
	RRh20	M	4	36.9%	9.53 × 10^6^	1.22 × 10^7^
	RWj20	M	4.4	ND	3.34 × 10^7^	9.59 × 10^7^
	RZj20	F	4.4	ND	1.61 × 10^7^	2.61 × 10^7^
	RZc20	F	7.1	25.0%	7.39 × 10^6^	1.18 × 10^7^
	RFb20	M	7.4	32.7%	3.40 × 10^7^	6.46 × 10^7^
	Mean		4.7	33.9%	3.40 × 10^7^	6.46 × 10^7^
Control	RQi20	M	3.7	34.9%	3.70 × 10^7^	1.00 × 10^8^
	RVb20	M	4	28.5%	3.77 × 10^6^	9.38 × 10^6^
	RQb20	F	4	23.3%	5.55 × 10^5^	9.89 × 10^5^
	RHb20	F	4.1	25.2%	1.99 × 10^6^	4.05 × 10^6^
	Mean		4.0	28.0%	1.08 × 10^7^	2.86 × 10^7^

### Impact of AZD5582 on latency reversal in infant RMs.

**FIG 2:**
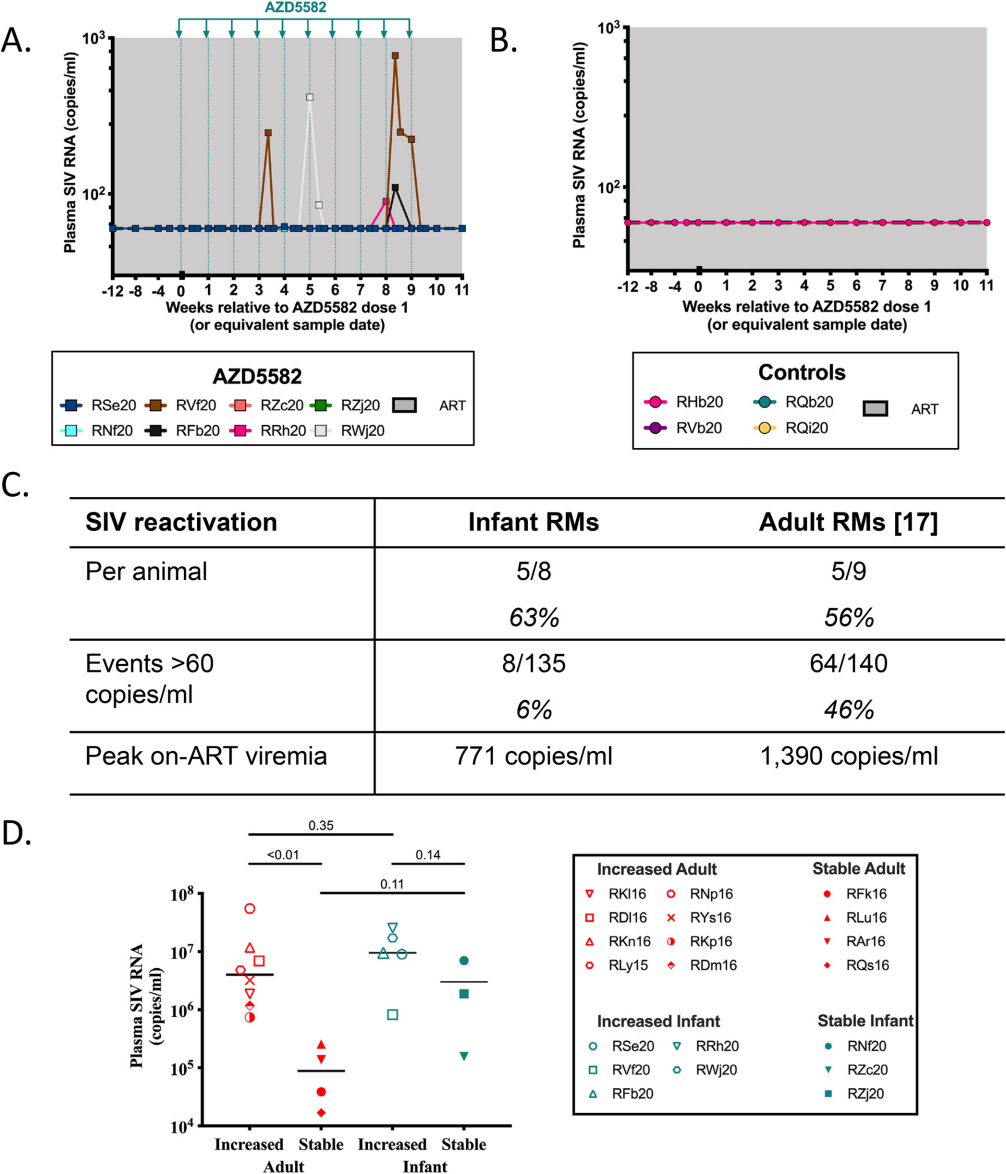
AZD5582 treatment and on-ART viremia in SIV-infected, ART-suppressed infant RMs. Longitudinal analysis of plasma SIV RNA levels during the intervention phase in (A) AZD5582 and (B) control groups. AZD5582 doses are indicated by green lines. The shaded area represents ART treatment. The dashed line represents the limit of detection of the assay. (C) Comparison of on-ART viremia in SIV-infected, ART-treated infant macaques and SIV-infected, ART-treated adult macaques. (D) Comparison of pre-ART plasma viral loads in adult and infant RMs that experienced on-ART viremia during AZD5582 treatment (increased) or remained stably suppressed throughout AZD5582 treatment (stable). The solid line represents the median. Experimental groups were compared using a two-sided Mann-Whitney test.

In this study, latency reversal was defined as a plasma SIV RNA level above 60 copies/mL during AZD5582 treatment in the presence of continued daily ART (or, “on-ART viremia”). The first instance of latency reversal was observed at 48 h after the 4th dose (Fig. 2A). On-ART viremia peaked at 771 copies/mL in RVf20 2 days post-dose 9. In total, at least one episode of on-ART viremia was observed in 5/8 RMs (63%) throughout treatment (Fig. 2A and C) in contrast with the durable viral suppression below 60 copies/mL observed in ART only controls (Fig. 2B). This is comparable to our previously published study in which 5/9 (56%) AZD5582-treated SIV-infected adult RMs experienced on-ART viremia (Fig. 2C) (17). Cell-associated SIV RNA was measured in peripheral CD4^+^ T cells before dose 1, 3dp-, and 2wp-AZD5582 treatment, and levels remained stable in both experimental groups (not shown). We note that 3/5 macaques that experienced latency reversal during AZD5582 treatment showed only a single episode of low-level on-ART viremia (range: 62 to 110 copies/mL). This low-level viremia contrasts, however, with the sustained suppression below 60 copies/mL in the 10 months before AZD5582 treatment in these 3 infants (Fig. 1B).

Out of 135 total viral load measurements performed over the AZD5582 treatment course in the 5 RMs that showed on-ART viremia, 8 total measurements (6%) were above the limit of detection of 60 copies/mL. This frequency of on-ART viremia episodes was lower than that observed in RM adults treated with AZD5582 (64/140, 46%) (17), despite similar overall percentages of responding animals (Fig. 2C).

SIV-infected adult RMs that exhibited on-ART viremia during AZD5582 treatment could be distinguished from those with stably suppressed viral loads during AZD5582 treatment by their significantly higher pre-ART viral loads (21). We investigated this association in the infants studied here but did not see a similar trend, although we were limited by the small sample size (Fig. 2D). Although the pre-ART viral loads of infants and adults with on-ART viremia were similar (*P* = 0.35), there was no significant difference in pre-ART viral loads between infants that responded to AZD5582 and infants that remained stably suppressed (*P* = 0.14). Interestingly, the infant RM with the most pronounced latency reversal (RVf20) also had the lowest pre-ART viral load from the group with on-ART viremia (lower than two of the stably suppressed infants). Together, these data highlight distinct age-related responses to this dosing regimen of AZD5582.

### Immunologic and virologic impact of AZD5582 in infant RMs.

**FIG 3:**
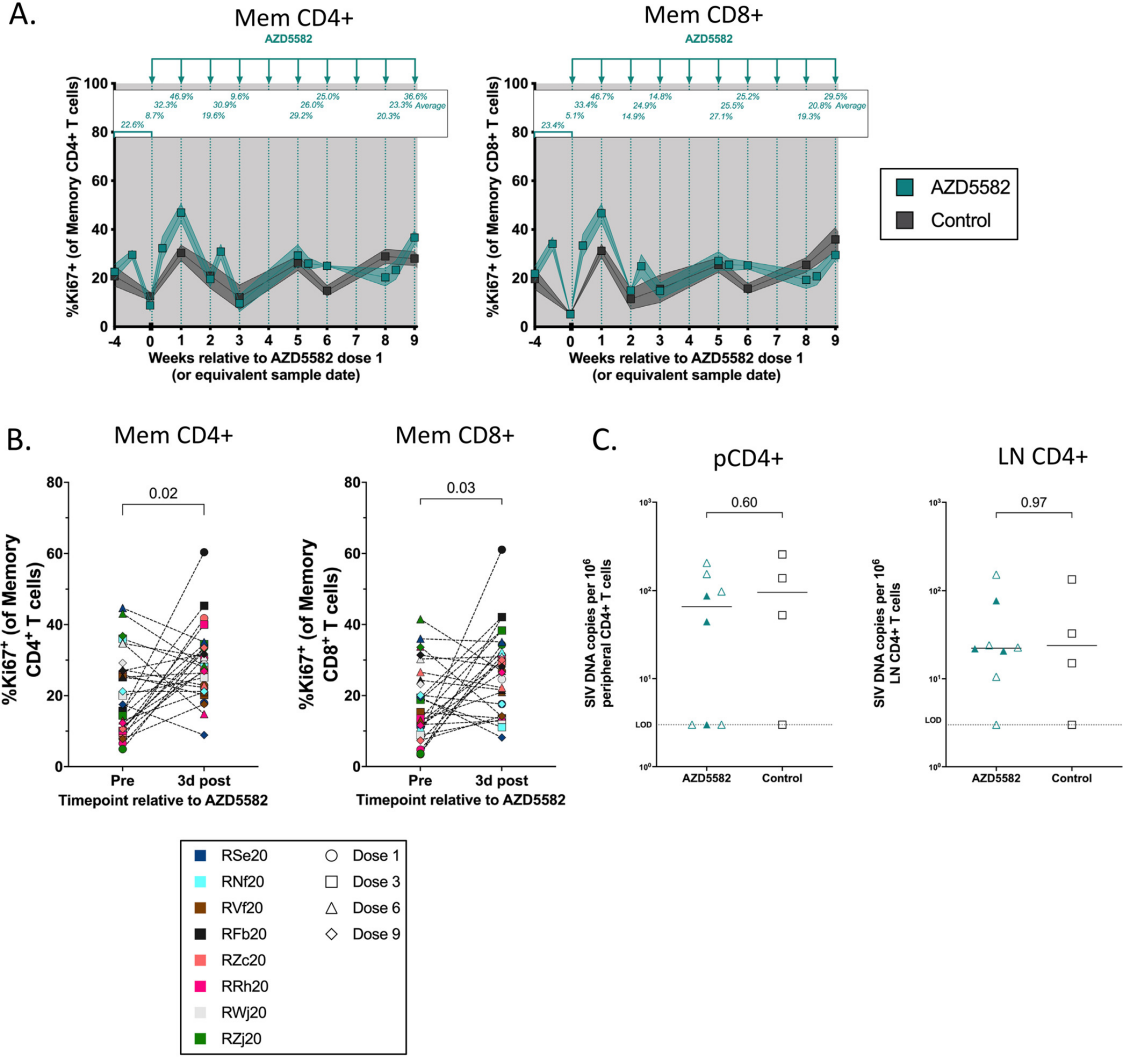
Immunologic and virologic response to repeated AZD5582 infusions in SIV-infected, ART-treated infant RMs. (A) Longitudinal analysis of Ki67 expression on memory CD4^+^ and memory CD8^+^ T cells in AZD5582-treated (teal, *n* = 8) and ART-only control (gray, *n* = 4) infant RMs. The mean of each time point is shown above except for baseline which is the mean of three time points. The shaded area represents the period of ART treatment and bars and shading represent mean ± SEM. (B) Frequency of Ki67^+^ memory CD4^+^ and CD8^+^ T cells immediately before and 3 days post-AZD5582 doses 1, 3, 6, and 9. Dose number is indicated by symbol shape and RM is indicated by symbol color. Statistical analysis was performed using a Wilcoxon matched-pairs signed-rank test. (C) SIV *gag* DNA levels in peripheral and LN CD4^+^ T cells after AZD5582 treatment (2 weeks post-dose 10) compared to control RMs sampled after a similar time on ART. Open symbols represent RMs that exhibited on-ART viremia and closed symbols represent animals that remained suppressed throughout the treatment period. The dashed line represents the limit of detection (LOD) for the assay. Statistical analysis was performed using a two-sided Mann-Whitney test.

The ncNF-κB pathway plays an important role in regulating T cell differentiation without causing broad systemic inflammation (22). We have previously observed a stimulatory effect on peripheral T cells following AZD5582 treatment specifically measurable through increased intracellular Ki67 expression (17). To further assess the effect of AZD5582 on infant RMs, flow cytometry was performed longitudinally on whole blood on the day of doses 1, 3, 6, and 9 and both 48 to 72h and 7 days after each of these doses. These time points were selected to allow longitudinal evaluation while remaining within blood volume constraints. Although natural fluctuations of Ki67 expression were observed throughout the experimental time course an increase in Ki67 expression in memory CD4^+^ and memory CD8^+^ T cells (identified as CD3^+^CD95^+^) was most prominent 7 days following dose 1 (Fig. 3A; 8.7% to 46.9% and 5.1% to 46.7%, respectively, in AZD5582 treated RMs compared with 5.3% to 31.2% and 12.5% to 30.4%, respectively, in control RMs). When three baseline measurements were used to account for natural variation, Ki67 expression increased by an average of 24.3% and 23.3% after dose 1 in memory CD4^+^ and CD8^+^ T cells, respectively, in treated RMs compared to 14.0% and 10.6% in control RMs (Fig. 3A). To assess the response to all doses in tandem, the expression of Ki67 in peripheral memory CD4^+^ and memory CD8^+^ T cells averaged 21.0% and 18.2%, respectively, at baseline and significantly increased to a mean of 28.4% and 26.5%, respectively, at 3-days post-AZD5582 (Fig. 3B; P = 0.02 and *P* = 0.03, respectively). We did not observe increases to other markers of activation, including HLA-DR and PD-1, following AZD5582 treatment. While we have previously shown that AZD5582 treatment alone did not consistently impact the size of the viral reservoir (17), we note that proliferation of CD4^+^ T cells (as suggested by increased Ki67 levels) may result in an expansion of infected cells. However, we found similar levels of cell-associated SIV DNA in CD4^+^ T cells isolated from the periphery and lymph nodes in AZD5582-treated infant RMs compared to ART-only controls (Fig. 3C).

### Pharmacokinetics of AZD5582 were altered in infant RMs.

**FIG 4:**
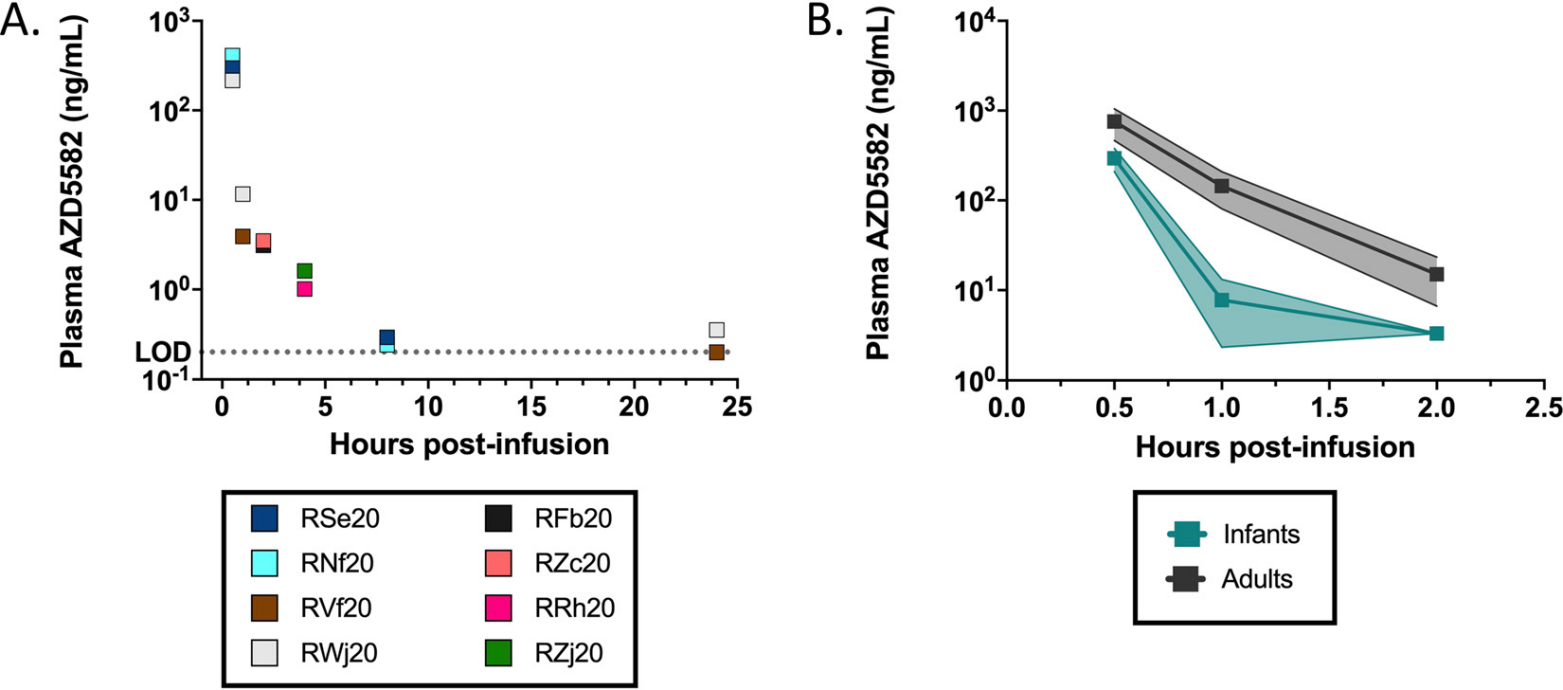
Pharmacokinetic assessment of AZD5582 in SIV-infected, ART-suppressed infant RMs. (A) AZD5582 (0.1 mg/kg) was administered by intravenous infusion and individual plasma concentrations are shown for the indicated time points (0.5h, *n* = 4; 1 to 24 h, *n* = 2). (B) Plasma concentrations of AZD5582 in infant (teal) and adult (gray) SIV-infected, ART-suppressed RMs for indicated time points. Bars and shading represent mean ± SD.

As a potential explanation for the dampened virologic response to AZD5582 observed in infant RMs, we investigated AZD5582 pharmacokinetics (PK) following the third dose using a sparse sampling design. Blood samples were collected immediately following infusion in 4 RMs and from 2 RMs per time point at 1 h, 2 h, 4 h, 8 h, and 24 h postinfusion. Plasma concentrations of AZD5582 in infants exhibited biphasic elimination with a rapid distribution half-life followed by a slower terminal elimination half-life of 9.9 h (Fig. 4A). Noncompartmental analysis (NCA) was conducted to determine pharmacokinetic parameters in infants and compared with available PK data from adult historical controls of SIV-infected, ART-suppressed rhesus macaques (*n* = 7) given matched doses of AZD5582 (Fig. 4B) (17). Infants exhibited 2.5- and 2.3-fold lower C_max_ and AUC_0-2h_, respectively (Table 2). Although corresponding data were not collected from adult macaques, we note that in infants the AUC_0-24h_ was calculated as 239 ng × hr/mL.

**TABLE 2:** Pharmacokinetic properties of AZD5582 in infants compared with adult SIV-infected, ART-suppressed RMs^*a*^

Age category	C_max_ (ng/mL)	AUC_0-2h_ (ng × hr/mL)	AUC_0-24h_ (ng × hr/mL)	t_1/2_ (hr)
Infant	294	223	239	9.9
Adult	802	512	ND	ND

a C_max_, maximum concentration; t_1/2_, terminal elimination halflife; AUC_0-2h_ and AUC_0-24h_, area under the curve from time zero to 2 or 24 h postinfusion, respectively. ND, not determined.

### Evaluation of ncNF-κB gene expression following AZD5582 treatment.

**FIG 5:**
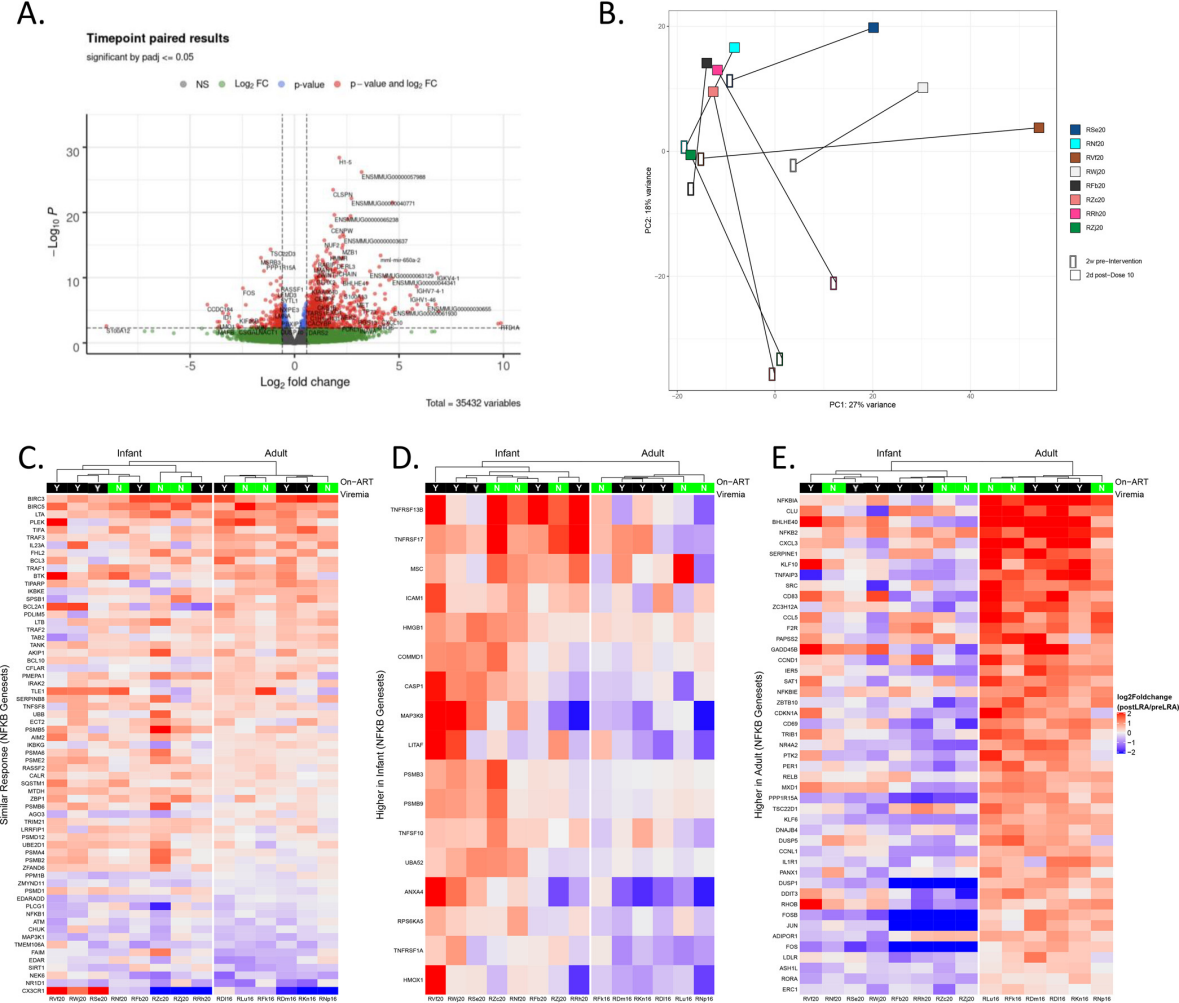
Gene expression changes in CD4^+^ T cells from peripheral blood of SIV-infected, ART-suppressed rhesus macaques before and after treatment with AZD5582. (A) Volcano plot showing genes upregulated or downregulated in peripheral CD4^+^ T cells following AZD5582 treatment compared to pre-AZD5582 treatment of SIV-infected, ART-suppressed infant RMs. Log_2_-fold change is represented on the *x*-axis and *P* value is represented on the *y*-axis. (B) Principal component (PC) analysis of the transcriptomes of CD4^+^ T cells from the peripheral blood before and after treatment with AZD5582. (C to E) Heat map of leading-edge genes that were differentially expressed after AZD5582 treatment and were (C) similar between infant and adult SIV-infected, ART-suppressed RMs, (D) higher in SIV-infected, ART-suppressed infant RMs, or (E) higher in SIV-infected, ART-suppressed adult RMs. The contrast depicted is the log_2_-fold change of each gene for each RM’s posttreatment sample relative to the pre-treatment values for peripheral CD4^+^ T cells. Annotation indicates the presence (black) or absence (green) of detectable on-ART viremia during AZD5582 treatment from pre-dose 1 to 3 d post-dose 10.

Treatment with AZD5582 in SIV-infected, ART-suppressed adult RMs upregulates key genes associated with signaling through the ncNF-kB pathway, rather than the canonical NF-κB (cNF-kB) pathway in CD4^+^ T cells (17). Here, we performed RNA-Seq in total CD4^+^ T cells isolated from the peripheral blood of AZD5582-treated infant macaques at baseline (pre-dose 1) and the end of the treatment period (post-dose 10). In total, 1,031 genes were identified as differentially expressed following AZD5582 treatment in infant RMs (855 upregulated and 176 downregulated) using a false discovery rate of 5% and a linear fold change of 50% (Fig. 5A). Principal-component analysis revealed a distinct effect of AZD5582 on gene expression in CD4^+^ T cells isolated from infant RMs 2 days post-dose 10 compared to peripheral CD4^+^ T cells from infants pre-AZD5582 (Fig. 5B).

Enrichment of NF-κB associated genes in AZD5582-treated infants was next compared with data generated from our prior adult macaque study, where RNA-Seq of peripheral CD4^+^ T cells was performed at the same time points (pre-AZD5582 and 2 days post-dose 10 of AZD5582) (17). The leading-edge genes in both groups of animals are shown in the heat maps in Fig. 5C to E, segregated by genes that were similarly changed in infants and adults (Fig. 5C), higher in infants compared to adults (Fig. 5D), and higher in adults compared to infants (Fig. 5E). *BIRC3* and *BIRC5* were both most significantly upregulated following AZD5582 in CD4^+^ T cells from AZD5582 treated infant and adult RMs (Fig. 5C). *BIRC3* encodes a cellular inhibitor of apoptosis 2 (cIAP2) and *BIRC5* (encoding survivin) also belongs to the IAP gene family, with both genes involved in the regulation of the cNF-κB and ncNF-κB pathways (23, 24). Genes identified as most upregulated in infant RMs but not adults include *TNFRSF13B* and *TNFRSF17*, two TNF-receptor superfamily genes that encode proteins that can initiate activation of NF-κB signaling (Fig. 5D) (25). Interestingly, *RELB* and *NFKB2*, hallmark ncNF-κB signaling genes, as well as *NFKBIA*, an inhibitory gene of the cNF-κB signaling pathway, were identified as significantly upregulated in CD4^+^ T cells isolated from adult RMs but not in infant RMs after AZD5582 treatment (Fig. 5E).

We did not identify a distinct gene expression profile that distinguished infants with on-ART viremia from those that did not show evidence of latency reversal during AZD5582 treatment (Fig. 5C and D). This result is consistent with our previous observations in adult RMs treated with AZD5582 (17) and indicates that *in vivo* administration of this IAPi can impact CD4^+^ T cell gene expression regardless of whether our definition of latency reversal was achieved. Even so, it is clear from these data that adult macaques demonstrate greater upregulation of ncNF-kB signaling genes as well as a greater frequency of latency reversal events during treatment with AZD5582 compared to infants, potentially due to the altered PK profile of AZD5582 in infants.

### *Ex vivo* evaluation of AZD5582 stimulation of infant CD4^+^ T cells.

**FIG 6:**
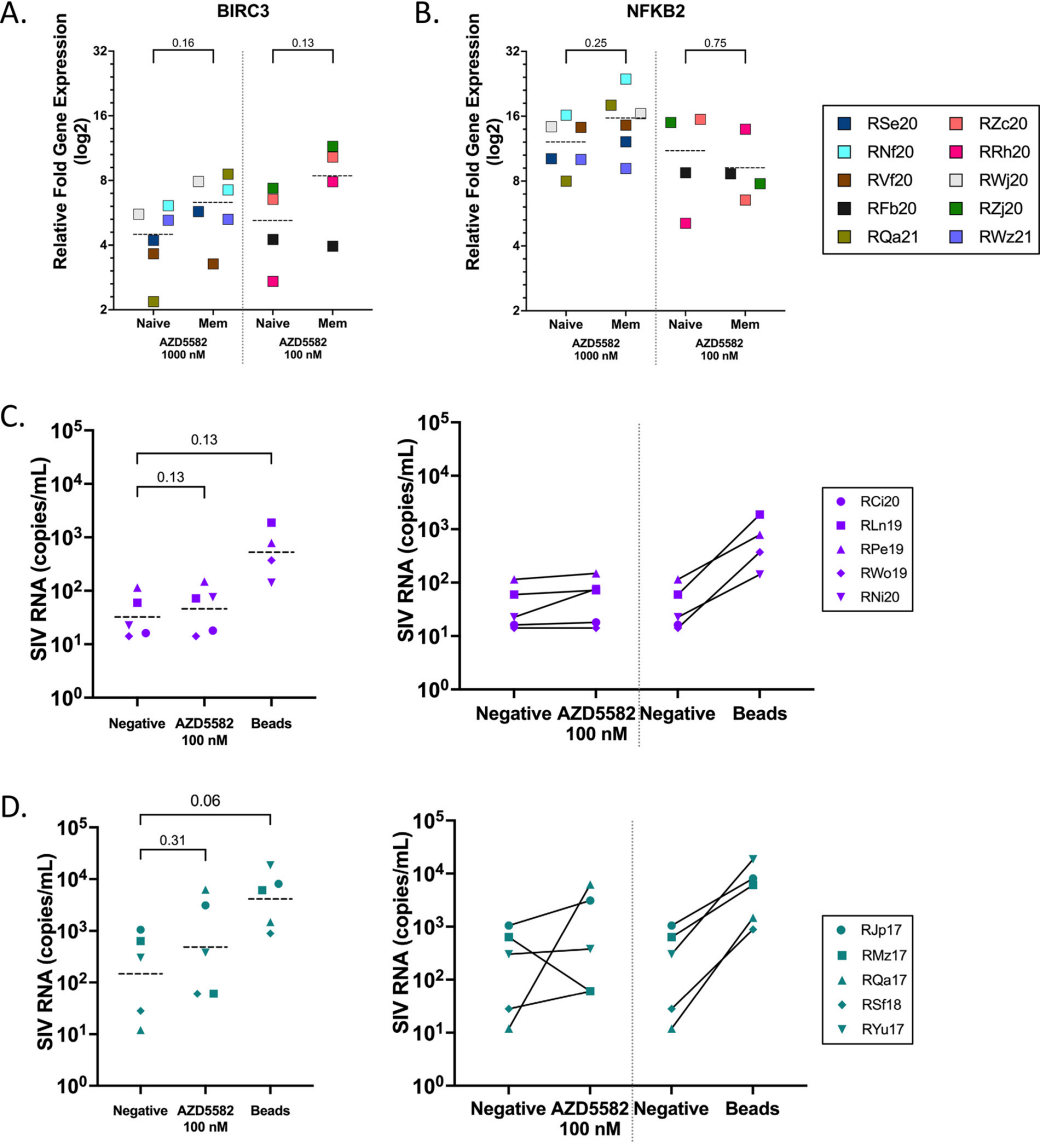
Induction of ncNF-kB genes and SIV RNA following *ex vivo* AZD5582 stimulation. (A and B) Naïve and Memory CD4^+^ T cells were sorted and treated with 1000 nM (*n* = 6) or 100 nM AZD5582 (*n* = 4) overnight. Cell lysates were analyzed by RT-PCR for (A) BIRC3 and (B) NFKB2, relative fold induction compared to housekeeping gene TATA box binding protein (TBP) with average expression in DMSO controls subtracted is shown. Symbols represent three technical replicates from a single run. The dashed line represents the mean. CD4^+^ subsets were compared using a Wilcoxon matched-pairs signed-rank test. (C and D) CD4^+^ T cells isolated from infant (C) and adult (D) RMs were purified and treated in the presence or absence of 100 nM AZD5582 with rhesus-specific anti-CD2/CD3/CD28-conjugated beads used as a positive control. Cells were treated for 9 days and SIV RNA expression was measured by qPCR in the supernatant on day 9. The dashed line represents the mean. Conditions were compared using a Wilcoxon matched-pairs signed-rank test (*P* < 0.05 was considered significant).

We have previously shown that naive CD4^+^ T cells are a major contributor to the viral reservoir in both SIV- and SHIV-infected infant macaques (10, 11). We were therefore interested to determine whether the dampened ART viremia that we observed *in vivo* was due to differential induction of ncNF-kB activation in naive and memory CD4^+^ T cells following AZD5582 treatment. It is well documented that activation of naive CD4^+^ T cells relies predominantly on cNF-κB and not the ncNF-κB pathway (22). However, AZD5582 targets intracellular components of the ncNF-κB pathway, including baculoviral inhibitor of apoptosis (IAP) repeat-containing 2 (BIRC2) and 3 (BIRC3), which together with another key component of ncNF-kB signaling, the NFKB inducing kinase (NIK), are equally expressed between naive and memory CD4^+^ T cells (26, 27). To confirm that the ncNF-κB pathway is activated following AZD5582 stimulation *ex vivo* in naive and memory CD4^+^ T cells, we purified naive (CD62L^+^CCR7^+^CD95-) and memory (CD95^+^) CD4^+^ T cells from the peripheral blood of two healthy and eight SIV-infected, ART-suppressed infant macaques. Sorted cells were stimulated with 100 or 1,000 nM AZD5582 for 24 h and RT-qPCR was performed to evaluate the expression of the ncNF-κB pathway genes *BIRC3* and *NFkB2* (Fig. 6A and B). When gene expression of BIRC3 or NFKB2 were compared in SIV-infected, ART-suppressed infants and healthy controls following stimulation with 1000 nM AZD5582 in naive and memory CD4^+^ T cells no significant difference was observed between the two groups. For this reason, all 1000 nM stimulations are represented together as a single group. No significant difference was observed in fold induction of *BIRC3* and *NFkB2* gene expression between naive and memory CD4^+^ T cells at 1000 nM (*P* = 0.16 and 0.25, respectively, *n* = 6) or 100 nM (*P* = 0.13 and 0.75, respectively, *n* = 4). These results indicate that both naive and memory CD4^+^ T cells have the potential to utilize the ncNF-κB pathway following AZD5582 stimulation despite the more modest gene expression changes observed in infants *in vivo* (Fig. 5).

Following confirmation of ncNF-κB pathway activation in infant naive and memory CD4^+^ T cells *ex vivo*, we next sought to evaluate SIV reactivation following *ex vivo* AZD5582 stimulation in CD4^+^ T cells isolated from SIV-infected, ART-suppressed infant RMs compared to CD4^+^ T cells from SIV-infected, ART-suppressed adult RMs. To do so, we isolated CD4^+^ T cells through negative selection from the peripheral blood of five SIV-infected, ART-suppressed adult RMs and five SIV-infected, ART-suppressed infant RMs. Cells were stimulated with either 100 nM AZD5582 or rhesus specific anti-CD2/CD3/CD28 beads (as a positive control) in the presence of ART (DTG+FTC) for 9 days. The supernatant was collected to quantify SIVgag RNA through qPCR as a measure of viral reactivation. All infant and adult cultures showed an increase in SIV RNA copies in the supernatant following bead stimulation compared to unstimulated wells (Fig. 6C and D). In the AZD5582 condition, only 1/5 infants showed increased SIV RNA expression compared to 3/5 adults (Fig. 6C and D). These data indicate that AZD5582 stimulation does not substantially induce virus reactivation in CD4^+^ T cells from infant RMs *ex vivo* and support the observed weaker latency reversal in infants compared to adults *in vivo*.

## DISCUSSION

This study provides insight into the use of latency-reversing agents to reactivate the viral reservoir in a pediatric model of HIV-1 infection and ART suppression. We first demonstrated the safety of repeated AZD5582 infusions and evaluated pharmacokinetics and pharmacodynamics following AZD5582 treatment. The IAPi AZD5582 induced on-ART viremia in 63% of infants, a number similar to what has previously been reported in adult RMs. However, only 6% of assays of plasma viremia throughout AZD5582 treatment in the infants that experienced on-ART viremia were above the limit of detection, perhaps related to the altered pharmacokinetic profile compared to adult macaques and resultant differential gene expression patterns. Although pre-ART viral loads served as an efficient predictor of on-ART viremia following AZD5582 treatment in adults, in our infants, pre-ART viremia was not a predictor of the presence or absence of latency reversal during AZD5582 treatment. Finally, through flow cytometry, we identified an increase in Ki67 expression in peripheral memory CD4^+^ and memory CD8^+^ T cells at 3 days post-dose compared to the pre-dose time point. Despite this transient activation of memory CD4^+^ T cells, we did not observe an expansion of infected cells in peripheral blood or lymph nodes.

Quantification of plasma AZD5582 concentrations revealed that weight-based dosing resulted in lower Cmax and AUC_0-2h_ for infants compared to adult RMs, which likely contributed to the altered response observed here compared to our previously published adult study (17). Pharmacokinetic processes differ between children and adults and, therefore, the same dosage of many drugs may not correspond to the same pharmacological effect (28). Metabolism, body composition, and gastrointestinal absorption fluctuate during development and need to be considered when determining an optimal dosing strategy in pediatric populations (29). The dose we used (0.1 mg/kg) is the highest dose tested in SIV-infected RMs to our knowledge, and we found it to be well-tolerated and safe in infants over 10 infusions. Thus, future efforts to maximize latency reversal in infants should involve testing a higher dose or longer infusion duration to identify an optimal AZD5582 dosing strategy for this age group. As the viral reservoir primarily consists of CD4^+^ T cells in tissues such as lymph nodes, evaluation of the concentrations of AZD5582 achieved in tissues will also be important to consider in future studies.

We found that the IAPi AZD5582 activates the NF-κB pathway in treated infant RMs as shown by transcriptomic analyses. IAP inhibitors target and activate the ncNF-κB pathway, which leads to activation of fewer host genes than the cNF-κB pathway, limiting systemic activation and likely increasing clinical tolerability (25). Through transcriptomic profiling, key ncNF-κB pathway genes, such as *BIRC3* and *BIRC5*, were identified as significantly upregulated in both treated infant and adult RMs following AZD5582 treatment. However, we did not observe increased expression of hallmark ncNF-κB signaling genes *RELB* and *NFKB2* nor the cNF-κB pathway inhibitor *NFKBIA*, unlike what we have previously reported in adult macaques.

It is well established that naive CD4^+^ T cells, the predominant subset in infants (10, 11), rely on the cNF-κB pathway for activation while the ncNF-κB pathway is more important in memory CD4^+^ T cells (25). However, we demonstrated that there is no significant difference between upregulation of both ncNF-κB genes *BIRC3* and *NFKB2* following *ex vivo* AZD5582 stimulation in naive and memory CD4^+^ T cells isolated from SIV-infected, ART-suppressed infants. It has also been shown that infected naive cells are less inducible than more differentiated CD4^+^ T cells *in vitro* (30). Furthermore, naive CD4^+^ T cells produce less infectious virus following multiple rounds of stimulation compared to memory CD4^+^ T cells *ex vivo* (31). Conflicting data on the inducibility of naive CD4^+^ T cells exist, however, with Zerbato et al. (32) reporting similar levels of virus production in naive and central memory CD4^+^ T cells following CD3/CD28 stimulation. In a study specifically investigating the reactivation potential of the pediatric reservoir, Dhummakupt et al. (33) suggested that the reservoir from perinatally HIV-1 infected children is less inducible than the reservoir from HIV-1 infected adults *ex vivo*. In the present study, we demonstrate that infant CD4^+^ T cells do not demonstrate substantial viral reactivation when stimulated with AZD5582 *ex vivo*. While dampened pharmacokinetics of ADZ5582 in infant RMs compared to adult RMs was likely a key contributor to our virologic findings *in vivo*, our results also complement a growing body of evidence for a lower inducibility potential of the pediatric viral reservoir, which also may impact the response to AZD5582 in infants.

This study has several limitations. The small size of infant RMs limits blood volume availability and biopsy specimen frequency, reducing our ability to perform extensive evaluative assays. Specifically, we were unable to perform ultrasensitive plasma viral load quantification, an assay with a much lower limit of detection compared to the 60 copies/mL limit used here. Although the ultrasensitive assay may have revealed a higher frequency of reactivation events under ART, it is expected that an effective LRA will induce a high-level on-ART viremia to permit clearance of virally infected cells and, as such, results from an ultrasensitive assay would likely not have altered our key findings. Additionally, through *ex vivo* stimulation with AZD5582, we demonstrated similar activation of the ncNF-κB pathway in naive and memory CD4^+^ T cells, but we were unable to evaluate the effect of *in vivo* AZD5582 treatment on specific CD4^+^ T cell subsets. Latency reversal may differ between naive and memory CD4^+^ T cells despite similar ncNF-κB activation levels. Additionally, infant macaques were infected with SIVmac251 while adult macaques were infected with SIVmac239, but both viral strains induce robust infection and reservoir formation. Thus, we do not believe this impacted our results. Finally, although not unexpected, AZD5582 alone did not impact the viral reservoir size estimated by CD4^+^ T cell-associated SIV DNA, but we acknowledge that we did not analyze the replication-competent or rebound competent viral reservoir here. Despite these limitations, we believe studies such as this one demonstrate the importance of investigating HIV-1 in a pediatric model to provide key preclinical data of pediatric HIV-1 cure interventions.

In conclusion, we demonstrated that the IAPi AZD5582 is safe and can activate the T cell compartment in SIV-infected, ART-suppressed infant RMs. This activation differed from that seen in adult RMs in terms of ncNF-κB gene expression and extent of latency reversal, which may be driven by both altered drug metabolism and lower inducibility of the pediatric viral reservoir. We hope that future studies optimize LRA dosing in pediatric preclinical models and incorporate the use of a “kill” agent to aid the immune system in clearing virally infected, reactivated cells to reduce the latent viral reservoir.

## MATERIALS AND METHODS

### Cell sorting.

For cell sorting, peripheral CD4^+^ T cells were first enriched by negative selection with the use of magnetic beads and column purification (nonhuman primate CD4^+^ T cell isolation kit; Miltenyi). Enriched CD4^+^ T cells were then stained with viability dye (Live/Dead Aqua) and previously determined volumes of the following fluorescently conjugated MAbs: CD3-AF700 (clone SP34-2), CD8-APC-Cy7 (clone SK1), CD95-PE-Cy5 (clone DX2), CD62L-PE (clone SK11), and CCR7-PE-Cy7 (clone 3D12) from BD Biosciences; CD4-BV650 from BioLegend. Sorted live CD3^+^CD8-CD4^+^ populations were defined as follows: naive cells, CD62L^+^ CCR7^+^ CD95-; and memory, CD95^+^. Sorting was performed on a FACSAria LSR II (BD Biosciences) equipped with FACSDiva software.

### Target gene RT-qPCR.

Naïve and memory CD4^+^ T cells were treated with 100 or 1000 nM AZD5582 for 24 h and then stored as a dry pellet until RNA extraction. Total RNA was isolated using the RNEasy Minikit (Qiagen) according to the manufacturer’s instructions. The following TaqMan primer-probe sets were sourced from Applied Biosystems: Rh02837734_m1 (BIRC3), Rh01028900_m1 (NFKB2), and Rh00427620_m1 (TBP). TaqMan-based quantitative PCR with reverse transcription (RT–qPCR; Fast Virus 1-Step Master Mix, Applied Biosystems) was used to amplify host genes of interest and acquire the signal on an Applied Biosystems 7500 Fast System (ThermoFisher). Gene expression was normalized to TATA box binding protein (TBP) and the comparative threshold cycle (Ct) method (ΔΔCt) was used for relative quantification of gene expression. Relative quantification was analyzed by ABI 7500 Software (v.2.3, Life Technologies).

### Animals and infection.

Twelve infant, Indian RMs (Macaca mulatta), with the exclusion of Mamu B*08- and B*17-positive animals, were enrolled in this study. The animals were born at the Yerkes National Primate Research Center (YNPRC) to dams housed in indoor/outdoor group housing. The infants were removed from the dams when they were approximately 2 weeks old and transferred to an indoor nursery, where they were housed in social pairs with either full contact or protected contact for the duration of the study. The infants were fed in accordance with the YNPRC standard operating procedures (SOPs) for NHP feeding. After being removed from the dam, infants were fed center-approved milk replacer (Similac Advance, OptiGro Infant Formula with Iron and/or Similac Soy Isomil OptiGro Infant Formula with Iron; Abbott Nutrition, Columbus, OH) until 14 weeks of age. Infants were provided softened standard primate jumbo chow biscuits (Jumbo Monkey Diet 5037; Purina Mills, St. Louis, MO) and a portion of orange starting between 2 to 4 weeks of age. As animals aged additional enrichment of various fresh produce items were provided daily. The animals were orally infected at 4 to 5 weeks of age with two consecutive doses of 10^5^ TCID_50_ (50% tissue culture infectious doses) of SIV_mac251_. Three infants required multiple weekly 2-dose challenges before successful infection (totaling up to three challenges). Eight historical controls that followed the same regimen were included in this study. Yerkes National Primate Research Center is accredited by both the U.S. Department of Agriculture (USDA) and by the Association for Assessment and Accreditation of Laboratory Animal Care (AAALAC). All animal procedures were performed in accordance with guidelines established by the Emory University Institutional Animal Care and Use Committee Guidelines and those set up by the NIH’s Guide for the Care and Use of Laboratory Animals, 8th edition.

### Antiretroviral therapy.

The 12 RM infants were treated with a potent three-drug ART regimen initiated at 4 weeks postinfection. The preformulation ART cocktail contained two reverse transcriptase inhibitors, 5.1 mg/kg tenofovir disoproxil fumarate (TDF) and 40 mg/kg emtricitabine (FTC), plus 2.5 mg/kg of the integrase inhibitor dolutegravir (DTG). This ART cocktail was administered once daily at 1 mg/kg via the subcutaneous route.

### Administration of AZD5582.

The IAPi AZD5582 was reconstituted to 0.4 mg/mL in 10% Captisol within 1 week of administration as previously described (17). Monkeys assigned to the experimental intervention group received 10 administrations of 0.1 mg/kg AZD5582 by intravenous (i.v.) infusion with an inline filter for 30 minutes every week for 10 weeks.

### Sample collection and processing.

EDTA-anticoagulated blood samples were collected regularly and used for a complete blood count, routine chemical analysis, and immunostaining, with plasma separated by centrifugation within 1 h of phlebotomy. PBMCs were prepared by density gradient centrifugation. Lymph node biopsy specimens were collected at indicated time points (Fig. 2A). Lymph nodes were ground using a 70-μm cell strainer. Cell suspensions were washed and immediately used for immunostaining or cryopreserved at −80°C until use.

### Immunophenotype by flow cytometry.

Multicolor flow cytometric analysis was performed on whole blood (WB) or cell suspensions using predetermined optimal concentrations of the following fluorescently conjugated monoclonal antibodies (MAbs). For WB T cell analysis the following MAbs were used: CD3-allophycocyanin (APC)-Cy7 (clone SP34-2), CD95-phycoerythrin (PE)-Cy5 (clone DX2), Ki67-AF700 (clone B56), HLA-DR-peridinin chlorophyll protein (PerCP)-Cy5.5 (clone G46-6), CCR7-fluorescein isothiocyanate (FITC) (clone 150503), CCR5-APC (clone 3A9), CD62L (clone SK11), and CD45-RA-PE-Cy7 (clone L45) from BD Biosciences; CD8-BV711 (clone RPA-T8), CD4-BV650 (clone OKT4), and PD-1-BV421 (clone EH12.2H7) from BioLegend; and CD28-ECD (clone CD28-2) from Beckman-Coulter. Flow cytometric acquisition and analysis of samples were performed on at least 100,000 events on an AURORA flow cytometer driven by the SpectroFlo software package (Cytek). Analyses of the acquired data were performed using FlowJo version 10.0.4 software (TreeStar).

### Plasma RNA and cell-associated DNA lysate viral quantification.

Plasma viral quantification was performed as described previously (12). The frozen cell pellet was lysed with proteinase K (100 μg/mL in 10 mM Tris-HCl pH 8) for 1h at 56°C. Quantification of SIVmac *gag* DNA was performed by quantitative PCR (qPCR) using the 5′ nuclease (TaqMan) assay with an ABI7500 system (PerkinElmer Life Sciences). The sequence of the forward primer for SIV_mac_ *gag* was 5′-GCAGAGGAGGAAATTACCCAGTAC-3′, the reverse primer sequence was 5′-CAATTTTACCCAGGCATTTAATGTT-3′, and the probe sequence was 5′-6-carboxyfluorescein (FAM)-TGTCCACCTGCCATTAAGCCCGA-6-carboxytetramethylrhodamine (TAMRA)-3′. 7.5 μL of cell lysate were mixed in a 50 μL reaction mixture containing 1× Platinum Buffer, 3.5 mM MgCl_2_, 0.2 mM dNTP, primers 200 nM, probe 150 nM, and 2U Platinum Taq. For cell number quantification, quantitative PCR was performed simultaneously for monkey albumin gene copy number. The sequence of the forward primer for albumin was F 5′-TGCATGAGAAAACGCCAGTAA-3′; the reverse primer sequence was 5′- ATGGTCGCCTGTTCACCAA-3′ and the probe sequence was 5′-AGAAAGTCACCAAATGCTGCACGGAATC-3′ (34). The reactions were performed on a 7500 real-time PCR system (Applied Biosystems) with the following thermal program: 10 min at 95°C, followed by 40 cycles of denaturation at 95°C for 15 s and annealing at 60°C for 1 min.

### Pharmacokinetics of AZD5582.

Plasma samples were collected from RMs over 24 h following the start of a single 30 min intravenous infusion of AZD5582 using a previously published (17). A high-performance liquid chromatography-mass spectrometry (HPLC-MS/MS) method with a dynamic range of 0.2 to 1000 ng/mL. Noncompartmental analysis (NCA) was performed using Phoenix64 WinNonlin v8.1 software using the sparse sampling function to derive mean PK parameters. The linear up-log down trapezoidal rule was used to calculate AUC. The terminal elimination rate constant (k_el_) was estimated by fitting a linear regression line on a semilog plot to the individual concentration data constrained to observations from 4 to 24 h post start of infusion and used to calculate terminal elimination half-life. One observation below the assay’s limit of quantification was imputed at ½ the lower limit of quantification (35).

### RNA-sequencing analysis.

RNA-sequencing (RNA-seq) analysis was conducted at the Yerkes Nonhuman Primate Genomics Core Laboratory (http://www.yerkes.emory.edu/nhp_genomics_core/). RNA was purified from 50,000 peripheral blood-derived CD4^+^ T cells purified by negative selection and lysed in 350 μL of RLT buffer at −80°C, using Qiagen Micro RNEasy columns, and RNA quality was assessed using an Agilent Bioanalyzer. Following this, 2 ng of total RNA was used as input for mRNA amplification using 5′ template switch PCR with the Clontech SMART-seq v4 Ultra Low Input RNA kit according to the manufacturer’s instructions. Amplified mRNA was fragmented and appended with dual-index bar codes using Illumina NexteraXT DNA library preparation kits. Libraries were validated by capillary electrophoresis on an Agilent 4200 TapeStation, pooled and sequenced on an Illumina HiSeq 3000 using 100-bp single reads at an average depth of 25 million reads. Alignment was performed using STAR version 2.7.3a (36) and transcripts were annotated using a composite reference of rhesus macaque (Mmul10 Ensembl release 100).

### Historical animal groups.

Data from an additional group of SIV-infected, ART-suppressed adult RMs treated with AZD5582 were used for comparative analysis (17). Nine Indian rhesus macaques, with the exclusion of MamuB*08+ and MamuB*17+ animals, were infected i.v. with 3 × 10^3^ TCID_50_ of SIV_mac239_ (*nef* open). Animals were treated with an identical ART regimen, as described above, initiated at 8 weeks postinfection. Animals remained suppressed for a similar time frame, over 1 year, as the infant macaques before AZD5582 treatment.

### *Ex vivo* viral RNA induction assay.

Viable cryopreserved PBMCs from SIV-infected, ART-suppressed infant and adult RMs collected 34 to 97 weeks post ART initiation were thawed, and CD4^+^ T cells were enriched by negative selection using the NHP CD4^+^ T cell isolation kit (Miltenyi Biotec). Enriched CD4^+^ T cells were rested overnight in R10 (RPMI + 10% FBS) and then cultured in the presence of 100 nM DTG, 1 μM FTC, and 50 IU/mL IL-2 at 2 × 10^6^ cells/mL and either 100 nM AZD5582 or rhesus-specific anti-CD2/CD3/CD28-conjugated beads (Miltenyi Biotec) as a positive control for 9 days. Cells plated ranged from 3.5 × 10^6^ to 6.5 × 10^6^ cells with the same cell input per condition for individual animals. One animal (RCi20) did not have sufficient cell availability for bead stimulation. Half of the culture medium was removed on days 3 and 6 and replaced with a fresh medium containing FTC, DTG, and IL-2. The viral RNA content of frozen cell-free culture supernatants collected on day 9 was determined using the SIV*gag* qPCR described above.

### Statistical analyses.

Statistical analyses were performed using GraphPad Prism Software (v.7 or v.8). *P *≤ 0.05 was considered statistically significant. To compare differences in pre-ART viral loads between experimental groups in Fig. 2D and SIV DNA levels in Fig. 3C a two-sided Mann-Whitney test was used. To test the statistical significance observed in Ki67 expression on memory CD4^+^ and CD8^+^ T cells in Fig. 3B, BIRC3, NFKB2 gene expression in Fig. 6A and B, and SIV RNA expression in Fig. 6C and D. A Wilcoxon matched-pairs signed-rank test was used. For RNA-seq analysis in Fig. 5, RNA-seq data were mapped to the NCBI Mmul10 assembly of the Indian rhesus macaque genome, and alignment was performed with STAR (v.2.7.3a) using Ensemble release 100 annotations as a transcript annotation and splice junction reference. Transcript abundance estimates were calculated internal to the STAR aligner using the algorithm of htseq-count (37). DESeq2 (38) was used for normalization and differential expression analysis. Gene set enrichment analysis (39) (GSEA), performed with the GSEA desktop module (available at https://www.broadinstitute.org/gsea/) and the Molecular Signatures Database (MSigDB), was used to determine pathway/gene set enrichment. Heat maps, volcano plots, and principal-component analysis plots were generated with the R (v 3.6.0) package ggplot2.

## References

[1] Global HIV & AIDS Statistics Fact sheet. 2020. https://www.unaids.org/en/resources/fact-sheet. Accessed October 6, 2020.

[2] Luzuriaga K, Mofenson LM. 2016. Challenges in the elimination of pediatric HIV-1 infection. N Engl J Med 374:761–770. 10.1056/NEJMra1505256. 26933850

[3] Finzi D, Hermankova M, Pierson T, Carruth LM, Buck C, Chaisson RE, Quinn TC, Chadwick K, Margolick J, Brookmeyer R, Gallant J, Markowitz M, Ho DD, Richman DD, Siliciano RF. 1997. Identification of a reservoir for HIV-1 in patients on highly active antiretroviral therapy. Science 278:1295–1300. 10.1126/science.278.5341.1295. 9360927

[4] Finzi D, Blankson J, Siliciano JD, Margolick JB, Chadwick K, Pierson T, Smith K, Lisziewicz J, Lori F, Flexner C, Quinn TC, Chaisson RE, Rosenberg E, Walker B, Gange S, Gallant J, Siliciano RF. 1999. Latent infection of CD4+ T cells provides a mechanism for lifelong persistence of HIV-1, even in patients on effective combination therapy. Nat Med 5:512–517. 10.1038/8394. 10229227

[5] Siliciano JD, Kajdas J, Finzi D, Quinn TC, Chadwick K, Margolick JB, Kovacs C, Gange SJ, Siliciano RF. 2003. Long-term follow-up studies confirm the stability of the latent reservoir for HIV-1 in resting CD4+ T cells. Nat Med 9:727–728. 10.1038/nm880. 12754504

[6] Whitney JB, Hill AL, Sanisetty S, Penaloza-MacMaster P, Liu J, Shetty M, Parenteau L, Cabral C, Shields J, Blackmore S, Smith JY, Brinkman AL, Peter LE, Mathew SI, Smith KM, Borducchi EN, Rosenbloom DI, Lewis MG, Hattersley J, Li B, Hesselgesser J, Geleziunas R, Robb ML, Kim JH, Michael NL, Barouch DH. 2014. Rapid seeding of the viral reservoir prior to SIV viraemia in rhesus monkeys. Nature 512:74–77. 10.1038/nature13594. 25042999 PMC4126858

[7] Goulder PJ, Lewin SR, Leitman EM. 2016. Paediatric HIV infection: the potential for cure. Nat Rev Immunol 16:259–271. 10.1038/nri.2016.19. 26972723 PMC5694689

[8] Del Prete GQ, Smedley J, Macallister R, Jones GS, Li B, Hattersley J, Zheng J, Piatak M, Jr, Keele BF, Hesselgesser J, Geleziunas R, Lifson JD. 2016. Short communication: comparative evaluation of coformulated injectable combination antiretroviral therapy regimens in simian immunodeficiency virus-infected rhesus macaques. AIDS Res Hum Retroviruses 32:163–168. 10.1089/aid.2015.0130. 26150024 PMC4761795

[9] Shytaj IL, Norelli S, Chirullo B, Della Corte A, Collins M, Yalley-Ogunro J, Greenhouse J, Iraci N, Acosta EP, Barreca ML, Lewis MG, Savarino A. 2012. A highly intensified ART regimen induces long-term viral suppression and restriction of the viral reservoir in a simian AIDS model. PLoS Pathog 8:e1002774. 10.1371/journal.ppat.1002774. 22737073 PMC3380955

[10] Mavigner M, Habib J, Deleage C, Rosen E, Mattingly C, Bricker K, Kashuba A, Amblard F, Schinazi RF, Lawson B, Vanderford TH, Jean S, Cohen J, McGary C, Paiardini M, Wood MP, Sodora DL, Silvestri G, Estes J, Chahroudi A. 2018. SIV persistence in cellular and anatomic reservoirs in ART-suppressed infant rhesus macaques. J Virol 92:e00562-18. 10.1128/JVI.00562-18. 29997216 PMC6146711

[11] Obregon-Perko V, Bricker K, Mensah G, Uddin F, Kumar M, Fray E, Siliciano RF, Schoof N, Horner A, Mavigner M, Liang S, Vanderford T, Sass J, Chan C, Berendam SJ, Bar K, Shaw GM, Silvestri G, Fouda G, Permar S, Chahroudi A. 2020. SHIV.C.CH505 persistence in ART-suppressed infant macaques is characterized by elevated SHIV RNA in the Gut and high abundance of intact SHIV DNA in naive CD4+ T cells. J Virol 95:e01669-20. 10.1128/JVI.01669-20. 33087463 PMC7944446

[12] Bricker KM, Obregon-Perko V, Uddin F, Williams B, Uffman EA, Garrido C, Fouda GG, Geleziunas R, Robb M, Michael N, Barouch DH, Chahroudi A. 2020. Therapeutic vaccination of SIV-infected, ART-treated infant rhesus macaques using Ad48/MVA in combination with TLR-7 stimulation. PLoS Pathog 16:e1008954. 10.1371/journal.ppat.1008954. 33104758 PMC7644092

[13] Margolis DM, Archin NM, Cohen MS, Eron JJ, Ferrari G, Garcia JV, Gay CL, Goonetilleke N, Joseph SB, Swanstrom R, Turner AW, Wahl A. 2020. Curing HIV: seeking to target and clear persistent infection. Cell 181:189–206. 10.1016/j.cell.2020.03.005. 32220311 PMC7896558

[14] Rasmussen TA, Lewin SR. 2016. Shocking HIV out of hiding: where are we with clinical trials of latency reversing agents? Curr Opin HIV AIDS 11:394–401. 10.1097/COH.0000000000000279. 26974532

[15] Zerbato JM, Purves HV, Lewin SR, Rasmussen TA. 2019. Between a shock and a hard place: challenges and developments in HIV latency reversal. Curr Opin Virol 38:1–9. 10.1016/j.coviro.2019.03.004. 31048093 PMC6819240

[16] Bricker KM, Chahroudi A, Mavigner M. 2021. New latency reversing agents for HIV-1 cure: insights from nonhuman primate models. Viruses 13:1560. 10.3390/v13081560. 34452425 PMC8402914

[17] Nixon CC, Mavigner M, Sampey GC, Brooks AD, Spagnuolo RA, Irlbeck DM, Mattingly C, Ho PT, Schoof N, Cammon CG, Tharp GK, Kanke M, Wang Z, Cleary RA, Upadhyay AA, De C, Wills SR, Falcinelli SD, Galardi C, Walum H, Schramm NJ, Deutsch J, Lifson JD, Fennessey CM, Keele BF, Jean S, Maguire S, Liao B, Browne EP, Ferris RG, Brehm JH, Favre D, Vanderford TH, Bosinger SE, Jones CD, Routy JP, Archin NM, Margolis DM, Wahl A, Dunham RM, Silvestri G, Chahroudi A, Garcia JV. 2020. Systemic HIV and SIV latency reversal via non-canonical NF-kappaB signalling in vivo. Nature 578:160–165. 10.1038/s41586-020-1951-3. 31969707 PMC7111210

[18] Meyers TM, Yotebieng M, Kuhn L, Moultrie H. 2011. Antiretroviral therapy responses among children attending a large public clinic in Soweto, South Africa. Pediatr Infect Dis J 30:974–979. 10.1097/INF.0b013e31822539f6. 21734620 PMC3193588

[19] Teasdale CA, Abrams EJ, Coovadia A, Strehlau R, Martens L, Kuhn L. 2013. Adherence and viral suppression among infants and young children initiating protease inhibitor-based antiretroviral therapy. Pediatr Infect Dis J 32:489–494. 10.1097/INF.0b013e31827e84ba. 23249913 PMC3624073

[20] van Dijk JH, Sutcliffe CG, Munsanje B, Sinywimaanzi P, Hamangaba F, Thuma PE, Moss WJ. 2011. HIV-infected children in rural Zambia achieve good immunologic and virologic outcomes two years after initiating antiretroviral therapy. PLoS One 6:e19006. 10.1371/journal.pone.0019006. 21552521 PMC3084269

[21] Dashti A, Waller C, Mavigner M, Schoof N, Bar KJ, Shaw GM, Vanderford TH, Liang S, Lifson JD, Dunham RM, Ferrari G, Tuyishime M, Lam CK, Nordstrom JL, Margolis DM, Silvestri G, Chahroudi A. 2020. SMAC mimetic plus triple-combination bispecific HIVxCD3 retargeting molecules in SHIV.C.CH505-infected, antiretroviral therapy-suppressed rhesus macaques. J Virol 94:e00793-20. 10.1128/JVI.00793-20. 32817214 PMC7565632

[22] Sun SC. 2017. The non-canonical NF-kappaB pathway in immunity and inflammation. Nat Rev Immunol 17:545–558. 10.1038/nri.2017.52. 28580957 PMC5753586

[23] Mahoney DJ, Cheung HH, Mrad RL, Plenchette S, Simard C, Enwere E, Arora V, Mak TW, Lacasse EC, Waring J, Korneluk RG. 2008. Both cIAP1 and cIAP2 regulate TNFalpha-mediated NF-kappaB activation. Proc Natl Acad Sci USA 105:11778–11783. 10.1073/pnas.0711122105. 18697935 PMC2575330

[24] Mohamed MS, Bishr MK, Almutairi FM, Ali AG. 2017. Inhibitors of apoptosis: clinical implications in cancer. Apoptosis 22:1487–1509. 10.1007/s10495-017-1429-4. 29067538

[25] Sun SC. 2012. The noncanonical NF-kappaB pathway. Immunol Rev 246:125–140. 10.1111/j.1600-065X.2011.01088.x. 22435551 PMC3313452

[26] Uhlen M, Fagerberg L, Hallstrom BM, Lindskog C, Oksvold P, Mardinoglu A, Sivertsson A, Kampf C, Sjostedt E, Asplund A, Olsson I, Edlund K, Lundberg E, Navani S, Szigyarto CA, Odeberg J, Djureinovic D, Takanen JO, Hober S, Alm T, Edqvist PH, Berling H, Tegel H, Mulder J, Rockberg J, Nilsson P, Schwenk JM, Hamsten M, von Feilitzen K, Forsberg M, Persson L, Johansson F, Zwahlen M, von Heijne G, Nielsen J, Ponten F. 2015. Proteomics. Tissue-based map of the human proteome. Science 347:1260419. 10.1126/science.1260419. 25613900

[27] Anonymous. 2021. Human protein atlas. http://www.proteinatlas.org/. Accessed September 15, 2021.

[28] Fernandez E, Perez R, Hernandez A, Tejada P, Arteta M, Ramos JT. 2011. Factors and mechanisms for pharmacokinetic differences between pediatric population and adults. Pharmaceutics 3:53–72. 10.3390/pharmaceutics3010053. 24310425 PMC3857037

[29] Shi R, Derendorf H. 2010. Pediatric dosing and body size in biotherapeutics. Pharmaceutics 2:389–418. 10.3390/pharmaceutics2040389. 27721364 PMC3967145

[30] Kulpa DA, Talla A, Brehm JH, Ribeiro SP, Yuan S, Bebin-Blackwell AG, Miller M, Barnard R, Deeks SG, Hazuda D, Chomont N, Sekaly RP. 2019. Differentiation into an effector memory phenotype potentiates HIV-1 latency reversal in CD4+ T Cells. J Virol 93:e00969-19. 10.1128/JVI.00969-19. 31578289 PMC6880164

[31] Kwon KJ, Timmons AE, Sengupta S, Simonetti FR, Zhang H, Hoh R, Deeks SG., Siliciano Jd, Siliciano Rf 2020. Different human resting memory CD4+ T cell subsets show similar low inducibility of latent HIV-1 proviruses. Sci Transl Med 12:eaax6795. 10.1126/scitranslmed.aax6795. 31996465 PMC7875249

[32] Zerbato JM, McMahon DK, Sobolewski MD, Mellors JW, Sluis-Cremer N. 2019. Naive CD4+ T cells harbor a large inducible reservoir of latent, replication-competent human immunodeficiency virus type 1. Clin Infect Dis 69:1919–1925. 10.1093/cid/ciz108. 30753360 PMC6853701

[33] Dhummakupt A, Rubens JH, Anderson T, Powell L, Nonyane BA, Siems LV, Collinson-Streng A, Nilles T, Jones RB, Tepper V, Agwu A, Persaud D. 2020. Differences in inducibility of the latent HIV reservoir in perinatal and adult infection. JCI Insight 5:e134105. 10.1172/jci.insight.134105. PMC7101150 31999647

[34] Bolton DL, Minang JT, Trivett MT, Song K, Tuscher JJ, Li Y, Piatak M, Jr, O'Connor D, Lifson JD, Roederer M, Ohlen C. 2010. Trafficking, persistence, and activation state of adoptively transferred allogeneic and autologous simian immunodeficiency virus-specific CD8(+) T cell clones during acute and chronic infection of rhesus macaques. J Immunol 184:303–314. 10.4049/jimmunol.0902413. 19949089 PMC2797565

[35] Beal SL. 2001. Ways to fit a PK model with some data below the quantification limit. J Pharmacokinet Pharmacodyn 28:481–504. 10.1023/A:1012299115260. 11768292

[36] Dobin A, Davis CA, Schlesinger F, Drenkow J, Zaleski C, Jha S, Batut P, Chaisson M, Gingeras TR. 2013. STAR: ultrafast universal RNA-seq aligner. Bioinformatics 29:15–21. 10.1093/bioinformatics/bts635. 23104886 PMC3530905

[37] Anders S, Pyl PT, Huber W. 2015. HTSeq–a Python framework to work with high-throughput sequencing data. Bioinformatics 31:166–169. 10.1093/bioinformatics/btu638. 25260700 PMC4287950

[38] Love MI, Huber W, Anders S. 2014. Moderated estimation of fold change and dispersion for RNA-seq data with DESeq2. Genome Biol 15:550. 10.1186/s13059-014-0550-8. 25516281 PMC4302049

[39] Subramanian A, Tamayo P, Mootha VK, Mukherjee S, Ebert BL, Gillette MA, Paulovich A, Pomeroy SL, Golub TR, Lander ES, Mesirov JP. 2005. Gene set enrichment analysis: a knowledge-based approach for interpreting genome-wide expression profiles. Proc Natl Acad Sci USA 102:15545–15550. 10.1073/pnas.0506580102. 16199517 PMC1239896

